# Audiological function in a group of adults following myringoplasty: an exploratory study in South Africa

**DOI:** 10.11604/pamj.2019.34.57.11714

**Published:** 2019-09-27

**Authors:** Katijah Khoza-Shangase, Namita Ramdin

**Affiliations:** 1 Department of Speech Pathology and Audiology, School of Human and Community Development, University of the Witwatersrand, Johannesburg, South Africa

**Keywords:** Audiology, hearing, HIV, myringoplasty, otitis media, tympanometry, tympanoplasty, South Africa

## Abstract

**Introduction:**

Chronic suppurative otitis media is a global middle ear disease with quality of life as economic implications, which are worse felt in low and middle income (LAMI) countries; thus the need for myringoplasty. This study aimed to explore audiological function in a group of adults following myringoplasty in South Africa, with an exploration of the possible influence of factors such as HIV/AIDS and type of surgical technique on hearing outcomes.

**Methods:**

Within a retrospective chart review research design, 41 participant files for a six-year period from two academic hospitals in Johannesburg, South Africa, were reviewed. Data were analysed using both descriptive and inferential statistics.

**Results:**

Participant files comprised of 16 males and 25 females between 18-63 years. Findings revealed that clinically, overall hearing improved post-operatively, as indicated by improved tympanometry findings, pure tone air-conduction and speech reception thresholds. Descriptively, the predictors of improved hearing outcomes post-operatively appeared to be HIV negative status and butterfly cartilage inlay surgery as a surgical technique adopted. Although clinically, hearing outcomes improved post-operatively at all air-conduction frequencies tested; these clinical improvements were only statistically significant at specific frequencies.

**Conclusion:**

Current findings provide useful initial evidence on the benefits of myringoplasty from the South African context; particularly because of the HIV/AIDS prevalence and its potential influence on middle ear disease and its management. Prospective efficacy studies with bigger sample sizes are recommended, with early identification strategies for middle ear disease to reduce the need for myringoplasty seriously considered bearing in mind the resource constraints.

## Introduction

Globally, hearing loss is the most common sensory deficit across the human race [1]. Eighty percent of deaf and hearing-impaired people live in low and middle-income countries where services are either totally absent or very limited [2]. WHO [1], further highlights that the major preventable causes of hearing impairment in these countries include middle ear infections. The burden of otitis media occurs overwhelmingly in these countries with almost nine times more cases reported if compared to developed countries [3]. Despite being the most prevalent disabling condition globally and one of the major contributors to the global burden of disease, hearing loss has historically been ignored on global health care agendas [4]. According to the WHO [5], it is “easily overlooked and underestimated” because it is not as “dramatic” as other health care conditions. It is therefore not surprising that hearing loss has been referred to as a silent epidemic. Hearing health care surveys confirm a global paucity of ear and hearing care services due to the limited numbers of available hearing health care professionals [2, 6]. A recent survey of countries in sub-Saharan Africa indicated that many of these countries do not have any audiology or otolaryngology services [2]. This shortage of hearing health care professionals is primarily due to a reported lack of government funding, professional and public awareness, and, most significantly, available training programs [6]. Only two African countries, for example, indicated having any training programs in audiology and many countries also indicated that they had no otolaryngology training programs [2].

Factors influencing health care in developing countries are numerous. South Africa has the largest number of people living with HIV/AIDS [7]. Disorders of the auditory and vestibular system, including otitis media, are often associated with HIV/AIDS [7, 8]. Otitis media as a global middle ear disease is reported to be as high as 11% in some African countries, with severe economic implications [9]. In South Africa, the high prevalence of otitis media has been attributed to factors such as overcrowding, inadequate housing, poor hygiene, not breastfeeding, poor nutrition, and high rates of nasopharyngeal colonisation with potentially pathogenic bacteria [10]. As with most infectious diseases, the burden of acute otitis media varies substantially across countries [11]. Studies have shown that the main differences in the manifestation of the burden of acute otitis media resides in the frequency of suppurative complications such as mastoiditis and meningitis and in the consequences such as hearing loss due to chronic suppurative otitis media as well as its management [12-14]. Management varies and presents a range of efficacy [15, 16]. Due to South Africa facing a number of challenges that complicate the progressive realization of access to health care [17], it can be suggested that not all patients are able to access treatment early and promptly thus leading to untreated otitis media which can result in permanent hearing loss.

South Africa has the highest income inequality globally and the gap between public and private health care, with regards to affordability and quality of service remains a great concern [17]. As a result of this inequality; majority of South Africa’s population can only afford access to government healthcare services. This places a huge demand on the government hospitals which results in long waiting periods for surgical procedures due to demand: capacity challenges [18]. Waiting lists for tympanostomy surgery may be as long as six months in these hospitals and this delay in intervention could potentially exacerbate the otitis media with effusion disease into chronic suppurative otitis media which then results in long term hearing loss as a consequence. Similar waiting lists exist for audiology services, therefore the often lack of pre and/or post-operative audiologic assessments, which are important for treatment efficacy evaluation. Literature suggests that up to 80% of the size of the eardrum perforation undergoes spontaneous closure, excluding need for surgery [19, 20]. Where spontaneous closure has not occurred, three principal indications for surgical intervention in the form of myringoplasty have been documented: (1) recurrent otorrhea, (2) desire to swim without wearing water proof in the ear, and (3) need to improve the conductive hearing loss resulting from a non-healing perforation [20]. Myringoplasty can improve hearing in many cases. Although a 90% success rate of surgery is frequently quoted for myringoplasty, the routine nature of the procedure, as well as the effect of many influencing factors on the success or failure of this procedure remains unresolved [20]. It has been suggested that factors such as the age of the patient; site of the perforation, size of the perforation, length of time that the ear has been dry for prior to surgery, the presence of infection at the time of surgery, as well as the status of the opposite ear may all be influencing factors affecting outcomes of myringoplasty [20, 21]. Several studies have reported that the most popular techniques for closure of a tympanic membrane perforation include either the temporalis fascia underlay approach or the butterfly cartilage inlay approach [21-26]; and these are the techniques investigated in this study as they were found to be the most common types of techniques performed at tertiary hospitals in Gauteng.

Clinical studies comparing outcomes of tympanic membrane reconstruction with fascia and cartilage show contradictory results [27], due to confounding variables such as revision surgery, variances in size and location of the perforation, a draining ear at the time of surgery, bilateral disease, ossicular discontinuity or cholesteatoma [25]. Hearing outcomes also vary considerably, with some studies indicating no significant difference in hearing outcomes post operatively while others do [26-28], with some studies from district hospitals in developing countries and remote indigenous communities demonstrating promising results [29-32]. However, some of these studies have not included audiological outcomes [31] or were limited by small sample sizes [32], while others have reported disappointing outcomes that are significantly poorer than those achieved at tertiary institutes in developed nations [33]. The reasons why outcomes of surgery for chronic otitis media remain poor in some developing countries and remote indigenous settings are not well understood [34-36]. Factors such as the nature of the disease, the availability of peri-operative care, the general health of the patient, age gender, size of perforation, and the level of surgical expertise have all been considered as potentially relevant [34, 36]. Literature reviewed has demonstrated that enough research has been done on the success rate and efficacy of myringoplasty surgery in terms of audiological improvement worldwide; more so in developed countries than in LAMI countries. Although it has been demonstrated that hearing does improve postoperatively in LAMI countries, the exact factors determining the outcomes of surgery and possible predictors of hearing improvement in this context remain unknown, thus the current study aimed at describing hearing function pre and post-operatively in a group of adults who had undergone Type 1 tympanoplasty surgery: myringoplasty at Academic Hospitals in Gauteng, South Africa. Specific sub-aims were to compare pre and post-operative hearing function; to explore the possible influence of HIV/AIDS on hearing outcomes post- surgery in those with HIV/AIDS; and to assess if the type of surgical technique has an influence on hearing outcomes post-surgery.

## Methods

### Research design

A retrospective chart review design was adopted [37], where medical, surgical and audiological records were reviewed.

### Participants and sampling

Through non-probability purposive sampling technique where participant files meeting specific inclusion criteria were included in the study [38]; a total of 41 participant files were included in the study (Table 1). Right, left or bilateral surgery was also documented as this affected the sample size per aim *(n)* in the data analysis. For participants who had bilateral surgery each ear was considered separately. So, the total sample size *(N)* comprised of 41 participant files, of these 8 participants had undergone bilateral surgery with each ear being considered separately; thus a total of 49 ears were investigated.

**Table 1:** Summary of the type and degree of hearing loss pre and post-operatively

Type	Pre	Post
Conductive hearing loss (CHL)	33	18
Mixed hearing loss (MHL)	16	13
Sensorineural hearing loss (SNHL)	0	5
Total no of hearing loss	49	36
**Degree**	**Pre**	**Post**
Normal	0	8
Mild	4	6
Moderate	19	12
Moderate-severe	15	6
Severe	4	2
Profound	7	2

KEY: **Pre**= pre-operatively, **Post**= post-operatively, **Normal**= hearing within normal limits, **Mild**= mild hearing loss, **Moderate**= moderate hearing loss, **Mod-sev**= moderate to severe hearing loss, **Severe**= severe hearing loss, **Profound**= profound hearing loss, **Total no of HL**= total number of hearing loss

Participants in the files selected had to meet the following criteria in order to be included in the study: 1) Participants needed to have undergone type 1 tympanoplasty primary surgery- myringoplasty at one of the two chosen tertiary academic hospitals. 2) Participants needed to be between 18-63 years of age as the study was focusing on adults; but the influence of presbycusis needed to be minimised as well. 3) HIV status of the participants needed to be known. 4) Each participant file needed to contain audiology testing results. The pre-operative results needed to have been within 3 months preceding surgery and post-operative results within 3-6 months following surgery. 5) Information on the type of surgery needed to be recorded in the files. Following a pre-study discussion with a senior oto-rhino-laryngologist on common surgical techniques at the research sites, two types of surgical techniques were included in the study i.e. butterfly cartilage inlay technique and fascia underlay technique.

### Data collection

The researcher documented the following biographical information and pre-post medical and audiological findings on a spreadsheet (Annex A).

**Annex 1:**
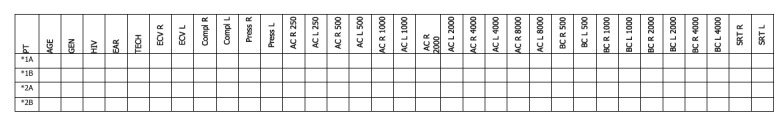
Data capturing form

***Biographical and medical information:*** 1) Age and gender of participant; 2) Ear in which surgery was performed; 3) Technique of surgery; 4) HIV status.

***Audiological information:*** 1) *Tympanometry results:* tympanometry results were classified according to the existing Jerger system for classification of types A-As, Ad, B, C and D with the type A regarded as normal middle ear functioning and all other types as abnormal, including Eustachian tube function findings where available [39]. 2) *Air-conduction and bone-conduction thresholds:* air-bone gaps (ABGs) at 500Hz, 1000Hz, 2000Hz and 4000Hz were calculated. According to Valente [39], normal hearing thresholds for adults are recorded at 25dB or less. Air-bone gaps are considered to be significant when greater than or equal to 10dB [39]. 3) *Speech reception threshold (SRT):* Speech reception threshold results for each ear were documented pre and post operatively.

### Data analysis and statistical procedures

Data were analysed using both descriptive and inferential statistics through the use of SPSS software. Descriptive analysis entailed a description of the demographical and medical presentation of the participants; and a clinical description of audiological data pre and post-operatively. Inferential statistics in the form of a paired t-test (two-tailed) to assess the changes in hearing function pre and post operatively as well as the influence of HIV status and type of surgical technique on hearing outcomes were used, set at a significance level of 0.05 (p-value); with surgery and HIV being independent variables ad post op outcomes dependent variable.

### Ethical considerations

The authors assert that all procedures contributing to this work comply with the ethical standards of the relevant national and institutional guidelines on human experimentation. Ethical approval from the University’s Medical Ethics Committee (Protocol number M121012) was obtained.

## Results

The sample comprised 16 males (39%) and 25 females (61%), with ages ranging from 18-63years with a mean age of 39.4 years were recruited. All participants of the selected files had undergone type 1 tympanoplasty, i.e. myringoplasty surgery in the last six years of the study being conducted. The sample was divided according to variables that would potentially affect hearing outcomes such as HIV status and type of surgical technique. Of the 41 participant files, 30 (73%) of the participants were HIV negative and 11 (27%) were HIV positive. Two types of surgical techniques were investigated. Of the 49 ears, 11 (22%) underwent the fascia underlay technique (F) while 38 (78%) underwent the butterfly cartilage inlay technique (C).

### What are the changes in hearing function pre and post type 1 tympanoplasty: myringoplasty?

#### Tympanometry

**Figure 1:**
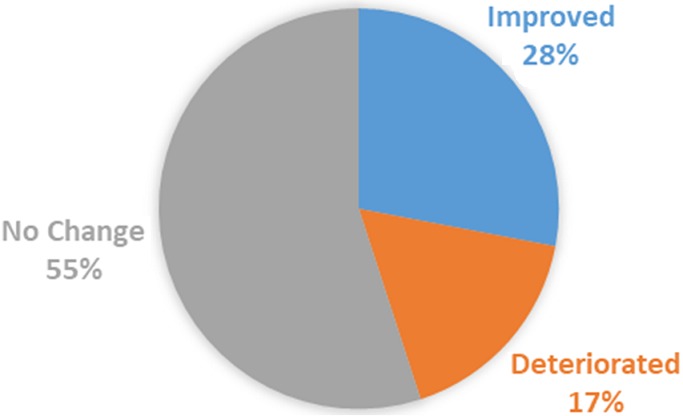
Tympanometry results post-operatively (n=36)

Figure 1 depicts tympanometry findings that indicate that a large majority of the sample showed no change in middle ear functioning through tympanometry testing. Of the 10 ears that showed an improvement in middle ear functioning; two were of participants who were HIV positive, eight were HIV negative. The majority of these participants underwent butterfly cartilage inlay technique of surgery (70%); which suggests that given the current sample size, this type of surgical technique seemed to descriptively yield the best middle ear functioning in HIV negative participants post-surgery.

#### Type of hearing loss

Descriptively, the type of hearing loss was analysed pre and post operatively (Table 1). Results revealed that the total number of ears with hearing loss (including conductive hearing loss and mixed hearing loss) pre operatively was 49 and this decreased post-operatively to 36. As depicted in Table 1, the total number of hearing loss decreased post-operatively suggesting that overall hearing (air-conduction and bone-conduction thresholds) improved post operatively in the total sample. This was particularly noted in participants with purely conductive hearing loss (CHL); without any sensory (inner ear) involvement. Five participants who had presented with mixed hearing loss pre-operatively had their conductive components of the hearing loss eliminated, leaving them with sensory loss postoperatively.

#### Degree of hearing loss

**Figure 2:**
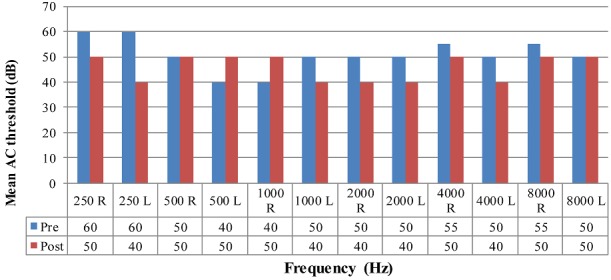
Mean air-conduction (AC) thresholds pre and post-operatively (dB)

**Figure 3:**
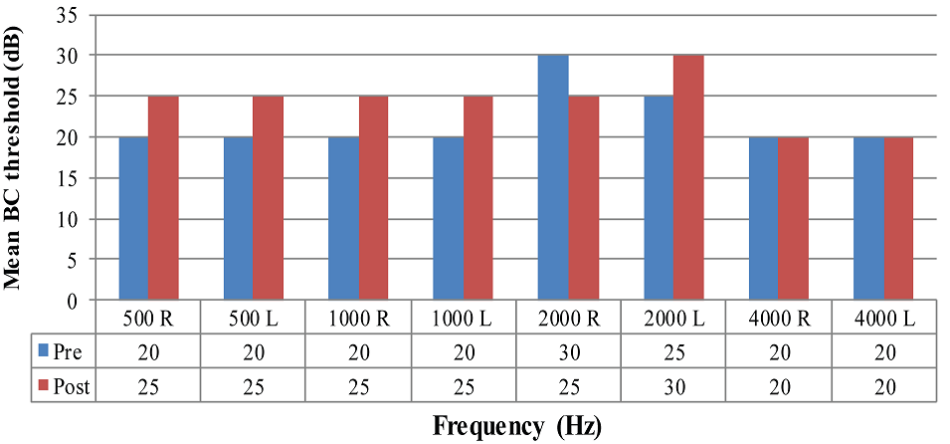
Mean bone-conduction (BC) thresholds pre and post-operatively (dB)

As depicted in Table 1, significant improvements in severity of hearing loss post-operatively were found. The most notable improvement was in the moderate-severe range; followed by the profound range of hearing loss. Post-operatively; at least 8 participants presented with hearing within normal limits; when they had not preoperatively. The mean air-conduction (AC) and bone-conduction (BC) thresholds were calculated pre and post-operatively at each frequency as depicted in Figure 2 and Figure 3. Descriptively, changes in hearing function in dB at different air-conduction frequencies was found to be clinically significant after surgery at 250, 500, 1000 and 2000Hz; frequencies most typically affected by conductive pathology. Figure 2 suggests that mean air-conduction thresholds decreased post-operatively at most frequencies implying that hearing ability improved overall post-operatively.

Figure 3 represents the mean bone-conduction results pre and post operatively. The results indicate that the mean pre thresholds were consistently lower than the mean post thresholds. This suggests that bone-conduction thresholds progressively worsened post-operatively; although this worsening in thresholds was overall not significant (<10dB). Hearing gain per frequency was calculated by subtracting the post-operative threshold from the pre-operative threshold (pre-post) for each frequency. If the difference between pre and post threshold is a positive value this suggests an improvement in hearing post-operatively. If the difference between pre and post is a negative value this suggests deterioration in hearing post-operatively as the pre threshold would have been the smaller value.

**Figure 4:**
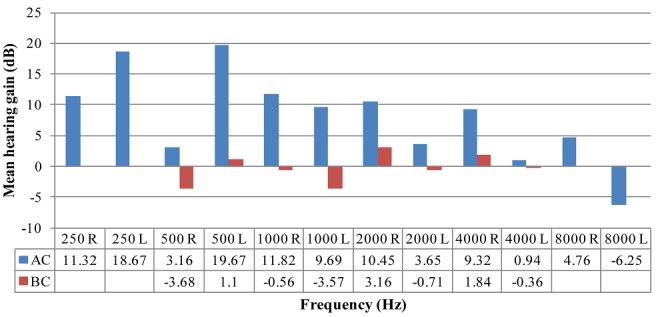
Mean hearing gain (dB) per frequency (Hz)

Figure 4 represents the mean gain per frequency in air-conduction and bone-conduction testing. Figure 4 displays the mean hearing gain in air and bone-conduction thresholds. It can be seen that for air-conduction thresholds there was an improvement in hearing (positive values) at all speech frequencies bilaterally. This suggests overall hearing improvement post-operatively for air-conduction thresholds. For bone-conduction, the mean hearing gain showed deterioration in hearing (negative values) at 500Hz, 1000Hz and 4000Hz. This suggests that the overall hearing deteriorated post-operatively for bone-conduction.

#### Air-bone gap (ABG)

**Figure 5:**
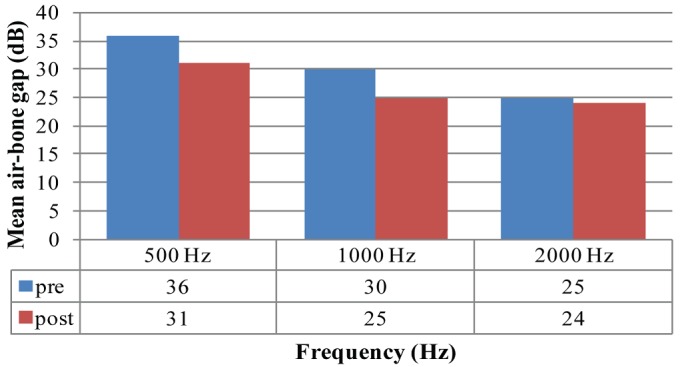
Mean air-bone gap (ABG) (dB) pre and post-operatively

Pre and post-operative air-bone gaps (ABGs) at 500Hz, 1000Hz and 2000Hz were calculated. The air-bone gap is the difference between the air-conduction threshold and the bone-conduction threshold. Figure 5 shows that the mean air-bone gap decreased post operatively; although not as significantly as anticipated; but the findings did suggest that the conductive component improved post-operatively overall. This correlated well with the earlier analysis on degree of hearing loss comparison pre and post-operatively.

### HIV status

Results in Table 2 show statistically significant differences at 250, 500 and 1000Hz at air-conduction frequencies in HIV negative participants but only at 250Hz in HIV positive participants. A paired t-test comparison in air-conduction outcomes in HIV positive participants to HIV negative participants showed no evidence of statistically significant differences across all air-conduction thresholds as shown in Table 2.

**Table 2:** Mean differences (pre-post) in hearing function pre and post-operatively by HIV status (air-conduction)

HIV Negative Patients (n=30)			HIV Positive Patients (n=11)				
AC frequency in Hz	Mean diff in hearing function (SD)	P-value	AC frequency in Hz	Mean diff in hearing function (SD)	P-value	Diff of Diff	P-value
**250**	15.60(22.65)	0.009*	**250**	13.9(17.64)	0.046*	1.71	0.854
**500**	17.40(23.02)	0.005*	**500**	7.78(18.22)	0.236	9.62	0.285
**1000**	11.04(19.30)	0.018*	**1000**	9.55(17.81)	0.106	1.49	0.838
**2000**	9.31(20.21)	0.060	**2000**	5.00(21.10)	0.450	4.31	0.585
**4000**	0(23.00)	1.051	**4000**	7.67(24.98)	0.255	-7.67	0.396
**8000**	-6.25(24.43)	0.267	**8000**	0(6.32)	1.000	-6.25	0.437

**KEY: SD**= standard deviation= standard error;

* **P-value**<0.05

Results presented in Table 3 show no statistically significant differences in mean change in hearing function in both HIV negative and positive participants at all frequencies tested by bone-conduction. Similarly, a comparison of mean differences in bone-conduction outcomes in HIV positive participants to HIV negative participants show no evidence of statistically significant differences across all frequencies as shown in Table 3.

**Table 3:** Mean differences (pre-post) in hearing function (dB) pre and post-operatively by HIV status (bone-conduction)

HIV Negative Patients (n=30)			HIV Positive Patients (n=11)				
Frequency in Hz	Mean diff (SD)	P-value	Frequency in Hz	Mean diff (SD)	P-value	Diff of Diff	P-value
500	-2.30(19.7)	0.73	500	-0.9(33.5)	0.929	-1.4	0.907
1000	-4.36(18.2)	0.423	1000	6.0(23.3)	0.437	-10.36	0.215
2000	-1.6(17.13)	0.732	2000	10.5(26.1)	0.214	-12.1	0.159
4000	1.8(22.2)	0.824	4000	6.8(27.6)	0.432	-5.0	0.602

**KEY: SD**= standard deviation= standard error; *P-value<0.05

#### Speech reception thresholds (SRTs)

Out of 49 ears only 27 ears (55%) had complete speech reception thresholds (SRTs) both pre and post operatively. Current findings indicate that there was an overall decrease in speech reception threshold (SRT) means post-surgery, suggesting improved speech reception thresholds post operatively. Twenty-three (85%) of the ears were of HIV negative individuals and four (15%) were from HIV positive individuals. Seven (26%) of the ears underwent fascia underlay (F) surgery, while 20 (74%) underwent butterfly cartilage inlay (C) surgery. Thus it can be suggested that in the current sub-sample (*n*=27) predictors for improved speech reception threshold results were HIV negative status and having undergone butterfly cartilage inlay surgery.

### Type of surgical technique

Results in Table 4 show statistically significant differences with regard to air-conduction mean change in hearing function in participants who had butterfly cartilage inlay surgery at 250, 500, 1000 and 2000Hz. On the other hand, no evidence of statistically significant difference in air-conduction mean change in hearing function was found in the participants who underwent fascia underlay surgical technique at all frequencies (Table 4).

**Table 4:** Mean differences (pre-post) in hearing function (dB) pre and post-operatively by type of surgical technique (air-conduction)

Cartilage (n=38)			Fascia underlay (n=11)				
AC frequency in Hz	Mean diff in hearing function (SD)	P-value	AC frequency in Hz	Mean diff in hearing function (SD)	P-value	Diff of Diff	P-value
250	20.21(23.1)	0.001*	250	15.3(20.1)	0.17	4.91	0.662
500	18.1(22.7)	0.002*	500	20.0(28.55)	0.231	-1.90	0.942
1000	14.44(21.0)	0.005*	1000	12.0(25.7)	0.434	2.44	0.836
2000	12.0(21.1)	0.016*	2000	4.0(33.1)	0.876	8.0	0.514
4000	9.2(24.83)	0.125	4000	-2.0(28.0)	0.887	11.2	0.399
8000	3.0(27.7)	0.767	8000	-10.0(12.0)	0.129	13.0	0.347

KEY: SD= standard deviation= standard error;

* P-value<0.05

Results in Table 5 show no statistically significant differences with respect to mean change in hearing function in participants who had butterfly cartilage inlay and fascia underlay surgeries at all frequencies.

**Table 5:** Mean differences (pre-post) in hearing function pre and post-operatively by type of surgical technique (bone-conduction)

Cartilage (n=38)			Fascia underlay (n=11)				
BC frequency in Hz	Mean diff in hearing function (SD)	P-value	BC frequency in Hz	Mean diff in hearing function (SD)	P-value	Diff of Diff	P-value
**500**	-2.76(27.9)	0.723	**500**	-2.5(31.0)	0.968	-0.26	0.985
**1000**	-0.6(24.7)	0.915	**1000**	3.75(11.23)	0.551	-4.35	0.736
**2000**	5.26(22.57)	0.344	**2000**	2.5(31.1)	0.972	2.76	0.835
**4000**	5.12(24.0)	0.486	**4000**	-1.3(38.9)	0.962	6.42	0.678

**KEY: SD**= standard deviation= standard error; *P-value<0.05

## Discussion

Current pure tone audiometry findings suggested an improvement in hearing outcomes post-surgery from a clinical audiological perspective. There was a decrease in the total number of hearing loss post operatively. These findings are consistent with those reported in previous studies [24, 20, 28, 35, 36, 40]. Furthermore, current findings revealed a significant decrease in the total number of conductive hearing loss post-operatively. Fifteen less participants presented with a purely conductive hearing loss, while five mixed hearing losses converted to sensory losses post-operatively. This implies success in surgical management for these participants; and also correlates with the evidence that states that conductive hearing loss generally improves post ear surgery in most cases [41].

As far as the degree of hearing loss was concerned, current findings showed significant improvements in severity of hearing loss post-operatively. The most notable improvement was in the moderate-severe range; followed by the profound range; with clinically significant improvements in low frequencies. Post-operatively, at least eight participants presented with hearing within normal limits; when they had not pre-operatively. These positive outcomes are consistent with those reported in previous studies [35, 36, 40]. Shresta and Sinah [35] analysed air-bone gaps and found that 78% of patients had their hearing gain exceeding 15dB after surgery while Shaikh *et al.* [36] report improved mean air-conduction thresholds post-operatively, even though the mean bone-conduction thresholds remained the same pre and post-operatively. The reason for this improvement may be due to restoration of the hearing mechanism post-operatively that improves air-conduction thresholds [20].

In the current study, the mean hearing gain for air-conduction and bone-conduction thresholds showed that air-conduction thresholds remained the same or improved and bone-conduction thresholds deteriorated overall. The improvements found were both clinically and statistically significant changes; which strengthens current findings. The statistically significant changes were at 250Hz, 500Hz, 1000Hz and 2000Hz where (*p* < 0.05); which implies improvement in functional hearing. These frequencies are the most important frequencies when evaluating hearing function as they are used to establish the degrees of hearing loss when the average threshold at three frequencies (500Hz, 1000Hz and 2000Hz) is calculated. The fact that in the current study, bone conduction deteriorated post-surgery could possibly be due to the type of surgical technique used in the majority of the participants; the butterfly cartilage inlay surgery; as opposed to the temporalis fascia underlay surgery adopted in Shaikh *et al.* [36] study. It must be noted though that bone-conduction thresholds are never looked at in isolation and form part of the diagnostic test battery for a comprehensive hearing assessment. Therefore, overall findings rather than isolated bone conduction thresholds from the current study were considered.

The improved speech reception threshold means post-operatively for the majority of the participants correlates well with the improved pure tone audiometry findings; and makes current findings generalizable to real functional use of hearing; instead of just pure tones. These findings correlate with those by Gerber *at al.* [28]. Furthermore, improved speech reception threshold results were in 85% participants who were HIV negative and 74% of those who had undergone butterfly cartilage inlay surgical technique; findings that correlate with earlier findings on pure tone audiometry. Current findings therefore, descriptively, seem to suggest HIV negative status and having undergone butterfly cartilage inlay surgery as predictors for improved hearing function following myringoplasty in the current sample. Geber *et al.* [28] also found that participants who underwent butterfly cartilage inlay surgery had better speech reception thresholds when compared to those who underwent fascia underlay surgery.

HIV status was found to be a possible variable that may have an influence on hearing outcomes post myringoplasty. These findings are of particular interest to the research and clinical community; as South Africa has the largest number of people living with HIV/AIDS. The mean change in air-conduction hearing was consistently lower in HIV negative participants at 250, 500, 1000 and 2000Hz. From 2000-8000Hz the mean change in hearing was higher in HIV negative participants and a subsequent decrease in mean hearing threshold was noted in HIV positive participants from 2000Hz. This correlates with trends in hearing loss in HIV positive participants found in the literature. For an example, Khoza-Shangase [42] found that 47% of HIV positive participants with clinical hearing loss had a tendency for sloping (high frequency) configuration of hearing loss; which is consistent with features typical of ototoxic hearing loss in participants with HIV. This correlates with the audiological results of the current study.

The better performance of HV negative patients is not surprising when one considers the various documented causes of hearing loss due to the immune-compromise that HIV positive patients present with [42, 43]. Firstly, the fact that HIV/AIDS presents the infected individual with primary causes of hearing loss in the form of the direct effects of the virus itself makes assessment of efficacy of treatments such as myringoplasty challenging. Secondly, the various opportunistic infections/conditions that HIV positive patients present with which are linked to auditory manifestations also need to be taken into account. In the current study, the various treatments that the patients were on; including ototoxic medications which have a direct impact on inner ear functioning could also be raised as possibly impacting current findings [44]. Lastly, within the South African context where universal treatment coverage for HIV/AIDS is far from being attained, treatment naïve related effects of HIV could be another factor with some of the participants in the current study.

When assessing the influence of butterfly cartilage inlay surgery on hearing outcomes post-operatively; statistically significant differences with regard to air-conduction mean change in hearing function were observed at 250Hz, 500Hz, 1000Hz and 2000Hz where (*p* < 0.05), therefore, suggesting that butterfly cartilage inlay surgery had an influence on air-conduction thresholds at specific frequencies. This correlates well with findings by Chhapola and Matta [26] where they claimed that cartilage perichondrium seems be an ideal graft material for tympanic membrane in terms of postoperative healing and improved acoustic properties as it can easily withstand negative middle ear pressure. Furthermore, they found statistical significant differences in all air-conduction thresholds [26]; as in the current study.

On the other hand, no evidence of statistically significant difference in air-conduction mean change in hearing function was found in the ears that underwent fascia underlay surgery at all air-conduction thresholds; suggesting that this type of surgical technique had limited influence on hearing outcomes post-operatively; although this is a preliminary finding since the sample size was small for this type of surgery. Although clinically, there was a difference in the post-operative findings between the two surgeries, these were not statistically significant at all air-conduction thresholds. This suggests that, statistically, the type of surgical technique has no influence on the hearing outcomes post-operatively. These findings highlight the importance of clinical evaluation and indicate that more detailed clinical conclusions must be drawn from a descriptive clinical evaluation rather than just on statistical analysis. Gerber *et al.* [28] compared hearing outcomes in participants who underwent fascia underlay and butterfly cartilage inlay and found no significant differences in air and bone-conduction thresholds. Mauri *et al.* [23] found that the audiometric results following inlay cartilage tympanoplasty or underlay tympanoplasty were similar; although inlay butterfly cartilage tympanoplasty presented with benefits of not requiring general anesthesia, was less expensive, and more comfortable to the patient - benefits which are important in a developing country where resources are limited. A single study conducted within the South African context by Becker and Lubbe [24] looked at the type of graft used (cartilage or temporalis fascia) as one of the prognostic factors that may influence outcomes of surgery and found that none of the prognostic factors assessed were statistically significant (*p* >0.05).

It must be noted that many of the studies reviewed did not use the same criteria to assess hearing outcomes when compared to that of the current study. There are several different kinds of myringoplasty techniques which are often adapted by surgeons according to their resources and clinical presentation of the patients; which differ from country to country. These variables make comparing findings from the current study to other similar studies challenging; hence the importance of interpreting current findings with these limitations in mind is recommended. Current findings should be interpreted within the identified methodological limitations. Firstly, the retrospective chart review design presented potential challenges related to data collection procedures and accuracy in data capturing. Secondly, the sample size as well as sample distribution limits generalization of current findings. The unequal distribution of participants between the two surgical techniques makes current findings preliminary in nature. Despite these limitations, current findings do raise important research, clinical management, policy, and planning future directions for this population within the South African context.

## Conclusion

The current study which aimed to explore the changes in hearing outcomes post myringoplasty and investigate the possible influence of HIV status and type of surgical technique on hearing outcomes post-operatively has implications for audiologists and otorhinolaryngologists working in South Africa. These findings can be used to guide audiologists in the management of patients’ pre and post myringoplasty surgery in terms of pre-testing and monitoring hearing function post-surgery; as well as in providing audiologists with possible variables that may influence hearing outcomes so that they can anticipate management protocols. This is particularly important in a resource constrained context where careful planning around health priorities is critical. This is also important because health budget allocation and spending is influenced by professionals proving the value of their service; and this is easily done with such efficacy studies. Current findings can also assist oto-rhino-laryngologists in anticipating hearing outcomes post myringoplasty surgery, which affects their decision making process in terms of type of surgical technique to be adopted. A study of this nature specifically investigating HIV status as a variable and its possible influence on hearing outcomes post myringoplasty surgery has never been conducted in the current context. Therefore, current findings may serve as a baseline for future studies which could specifically focus on HIV as an influencing factor; with strict controls of possible confounding variables which were not controlled for in the current study; since this was an exploratory study.

### What is known about this topic

- Extensive evidence exists on the success rate of myringoplasty in relation to functional gain in hearing post-surgery worldwide;
- International evidence exists on the efficacy of myringoplasty surgery in terms of audiological improvement worldwide;
- It is known that there is paucity of evidence within the developing world context, particularly from Africa.

### What this study adds

- Findings from this study indicating clinical improvement in hearing outcomes post-myringoplasty from a LAMI country, consistent with international studies, add to the surgical efficacy evidence from this context;
- The fact that HIV status was found to be a possible variable that may have an influence on hearing outcomes post myringoplasty is an important addition to the body of evidence;
- The importance of efficacy studies looking at both clinical and statistical significance of findings in their analysis was highlighted by the current study.

## Competing interests

The authors declare no competing interests.
